# Morphology and Function of Red Blood Cells in COVID-19 Patients: Current Overview 2023

**DOI:** 10.3390/life14040460

**Published:** 2024-04-01

**Authors:** Friedrich Jung, Philippe Connes

**Affiliations:** 1 Institute of Biotechnology, Molecular Cell Biology, Brandenburg University of Technology Cottbus-Senftenberg, 01968 Senftenberg, Germany; 2 Laboratory LIBM EA7424, Team “Vascular Biology and Red Blood Cell”, University of Lyon I, 69500 Lyon, France; philippe.connes@univ-lyon1.fr

**Keywords:** COVID-19, SARS-CoV-2, red blood cell, deformation, aggregation, endothelial cell

## Abstract

In severe cases, SARS-CoV-2 infection leads to severe respiratory failure. Although angiotensin-converting enzyme 2 (ACE2) receptors are not expressed in red blood cells, SARS-CoV-2 can interact with red blood cells (RBCs) via several receptors or auxiliary membrane proteins. Recent data show that viral infection causes significant damage to the RBCs, altering their morphology, deformability, and aggregability. Loss of RBC deformability and/or increased aggregability favors the development of thrombotic processes in the microcirculation, as has been described to occur in COVID-19 patients. In addition, many patients also develop systemic endotheliitis associated with generalized coagulopathy. This manifests itself clinically as obstructive microthrombi in the area of the medium and smallest vessels, which can affect all internal organs. It is thought that such changes in the RBCs may contribute to the microangiopathy/microthrombosis associated with COVID-19 and may result in impaired capillary blood flow and tissue oxygenation.

## 1. Introduction

COVID-19 is an acute respiratory infection caused by Severe Acute Respiratory Syndrome Coronavirus 2 (SARS-CoV-2). SARS-CoV-2 has spread rapidly around the world [1]. The first recorded symptoms of COVID-19 were respiratory symptoms with shortness of breath due to typical lung involvement [2,3]. A cytokine storm (interferon-α, and -γ, interleukins, tumor necrosis factor α, and tumor growth factor β), which can lead to a systemic inflammatory reaction, was thought to be the cause [4,5]. Clinical manifestations of such inflammatory responses include gastrointestinal, cardiovascular, neurological, dermatological, hematological, and musculoskeletal disorders [6,7]. Common symptoms of these disorders are microcirculatory disturbances including microthromboses, which have been described in various organs [8,9,10,11,12,13].

However, undisturbed microcirculation is a *conditio sine qua non* for an adequate supply of oxygen and nutrients to the tissue, with simultaneous removal of metabolic intermediates and end products. Red blood cells (RBCs)—as oxygen carriers—are the most important cellular components of the blood for homeostasis, as they are of paramount importance for the prevention of hypoxia and for functional microcirculation. Under physiological conditions, RBCs ensure an adequate supply of oxygen to the tissues while simultaneously removing carbon dioxide (CO_2_). Oxygen uptake into the RBCs occurs in the lungs, where intra-erythrocytic binding to hemoglobin, the essential protein for oxygen transport, takes place. The bound oxygen is then distributed throughout the organism via the bloodstream according to local demand.

Oxygen deficiency can be caused by a reduction in the diameter of blood vessels—in the worst case, by occlusion—or by changes in the fluidity of the blood. Rheological phenomena can lead to disturbances in microvascular perfusion. In the acute phase of COVID infection, the C-reactive protein and the fibrinogen concentration are significantly increased [14,15], which leads to an increase in plasma viscosity and thus to a slowing of blood flow in the microcirculatory pathway [16]. This is intensified by the rigidification of the erythrocytes [17], which further reduces the capillary passage [18]. The physiological deformability of RBCs allows them to enter very small capillaries (the physiological diameter of human RBCs is around 8 µm, while the smallest capillaries are around 3 µm) and to pass through capillaries [19]. Pathophysiological changes in the morphology and function of erythrocytes are known to occur in various diseases such as arterial occlusive disease, diabetes, sickle cell anemia or malaria. A rigidification of erythrocytes and an increased tendency to aggregate have now been observed in COVID-19 patients [17,20,21,22].

In addition, the platelets are displaced into the plasma zone near the vessel wall, ultimately promoting a prothrombotic state [23]. The significant decrease in blood flow through the microcirculation also leads to a decrease in shear forces in the venules, resulting in the aggregation of erythrocytes into rouleaux or clot aggregation [24,25], which is exacerbated by the reduced release of NO from endothelial cells [26].

It has been hypothesized that SARS-CoV-2 may interact with RBCs, although ACE2 receptors are not expressed in red blood cells [27]. However, several receptors or membrane proteins (including CD147, NRP-1, CD26, AGTR2, Band3, KREMEN1, ASGR1, ANP, TMEM30A, CLEC4G, and LDLRAD3) or the G protein-coupled receptor 78 (GRP78) [28] have been reported to act as entry receptors for SARS-CoV-2 [29]. Erythrocyte band 3 protein expression of erythrocytes may mediate binding to the S protein of SARS-CoV-2, which may then cause a change in oxygen transport with the development of hypoxia [30,31,32]. However, this hypothesis is based on *in silico* calculations and the interpretation of the results has been critically discussed by others [33]. On the other hand, the SARS-CoV-2 ORF8 protein can bind the porphyrin part of hemoglobin at the β1 chain, which can lead to functional impairment of hemoglobin or even hemolysis, which would reduce the oxygen transport capacity [34,35].

Typical signs of sepsis-like cold extremities have been reported, indicating a microcirculatory dysfunction [8,20], which could be due to hypercoagulability and blood hyperviscosity as reported by several authors [18,30,31,32]. Therefore, RBCs are of particular interest in the pathophysiology of COVID-19.

## 2. Influence of SARSCoV-2 on Red Blood Cell Function

Several mechanisms of interaction between SARS-CoV-2 and RBCs [31] can be considered. An extremely limited oxygen supply caused by dysfunctional RBCs may occur, which could contribute to the development of hypoxia-induced multi-organ damage in critically ill COVID-19 patients [32,36,37].

Berzuini et al. hypothesized that the dysfunction of RBCs is the result of immunological processes or physical damage to cells due to COVID-19 microangiopathy [21]. Rigid RBCs complement activation together with increased fibrinogen levels, and D-Dimer concentrations observed in COVID-19 may contribute to the formation of RBC aggregates and, possibly, to the microvascular thrombosis described in cases of COVID-19 [21]. Vianello’s group reported that hospitalized patients with COVID-19 frequently showed abnormal RBC morphology. In particular, spiculated cells are known to reflect an impaired protein and lipid membrane composition [22]. The degree of spiculation correlated with the severity of the viral infection. Therefore, Marchi et al. described the peripheral blood smear as a possible additional prognostic tool for COVID-19 [22]. The formation of spherocytes, schistocytes, stomatocytes, knizocytes, and mushroom-shaped RBCs has also been described [22,38]. Such morphologies are often associated with a reduction in RBC deformability [39].

Increased oxidative stress has also been reported in patients with COVID-19—as with many respiratory viral infections—which may promote inflammation, and activate apoptotic pathways and the innate immune response, leading to disturbed blood flow regulation and ultimately to tissue damage, possibly progressing to multi-organ dysfunction or failure [4,9,36]. RBCs may also be the target of this increased oxidative stress. High levels of reactive oxygen species (ROS) can damage the RBC membrane through lipid peroxidation and protein oxidation processes [40,41,42] and promote RBC senescence through calcium accumulation and phosphatidylserine exposure [40]. The subsequent loss of membrane elasticity and RBC deformability may then affect the ability of RBCs to deliver their oxygen to tissues and favor hypoxia and thrombotic events [43].

Recent studies revealed that the RBC deformability from COVID-19 patients was significantly lower than that of the control group [17,42,44,45], confirming previous findings [17]. Szewczykowski et al. reported that RBCs from COVID-19 patients exhibited reduced deformability, which could hinder their passage through the capillary system and reduce oxygen transportation through the body [44]. Recktenwald et al. observed an impairment of RBC shape in COVID-19 samples. These changes were reversible when the COVID-19 RBCs were suspended in the plasma of healthy volunteers [45]. Conversely, healthy RBCs resemble COVID-19 RBCs when suspended in COVID-19 plasma. Taken together, these findings indicate that the plasma of patients with COVID-19 contains some molecules that may alter the physical properties of RBCs. RBCs from COVID-19 patients were found to have increased levels of glycolytic intermediates, oxidation and fragmentation of ankyrin, α-spectrin, and the N-terminal cytosolic domain of band 3, which may explain the observed decreased deformability of RBCs [37]. This may be enhanced by the abundant presence of SARS-CoV-2 in the interior of erythrocytes, which may increase its internal viscosity, thereby decreasing RBC deformability [46]. Human erythrocytes with 1 × 10^6^ GPA molecules, the major sialoglycoprotein in human RBCs, can bind 1.5 × 10^5^ virions, with a maximum uptake within 1–2 h [47].

In addition, because the N-terminus of band 3 stabilizes deoxyhemoglobin and regulates the oxygen unloading and metabolic rewiring towards the hexose monophosphate shunt, COVID-19 RBCs may be less able to respond to variations in hemoglobin oxygen saturation. Structural changes in proteins together with membrane lipid remodeling may also impair oxygen transport to tissues [37].

RBC aggregation of has also been described to be markedly increased in COVID-19 patients [20]. *In vitro*, a strong correlation was found between plasma fibrinogen concentration and RBC hyperaggregation. In addition, RBC aggregation correlated positively with the clot firmness, and negatively with the clot formation time.

**Figure 1:**
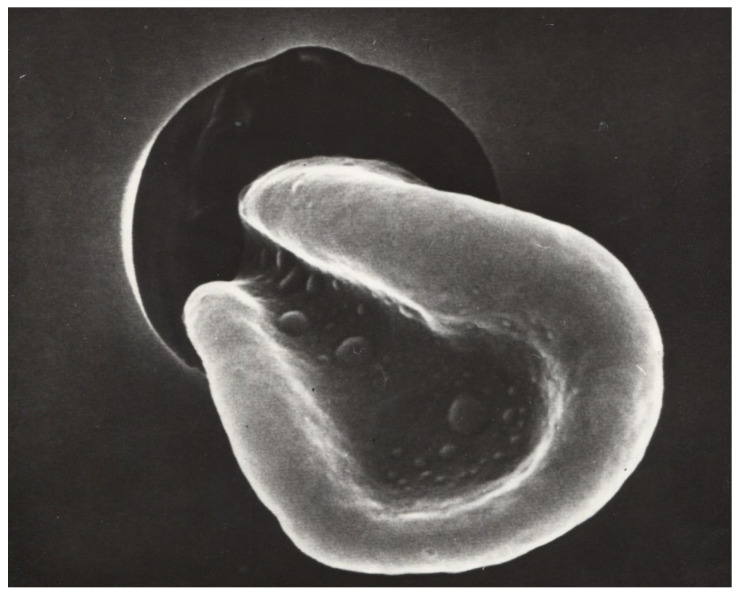
A red blood cell passing through a capillary under massive deformation (secondary electron microscopy, Dr. Hans Günther Roggenkamp [51]). magnification 1:1500.

The influence of the different hemorheological parameters on the velocity of red blood cells in human capillaries cannot be quantified independently of each other [18], partly because of the large variability in the diameter of human capillaries, with values ranging between 3 and 15 μm in healthy subjects. In patients—depending on the disease—the capillary diameters can reach up to 50 μm (e.g., giant capillaries in patients with scleroderma [43]). This is a very wide range in terms of the role of different rheological parameters of blood in capillaries. In small capillaries, plasma viscosity determines microvascular perfusion because the absolute capillary hematocrit is about 10–20%; thus, capillary blood viscosity approaches the same level as plasma viscosity [48]. This is true as long as the capillary diameter is larger than the RBC diameter [49,50]. When the capillary diameter becomes smaller than the RBC diameter (8 µm), RBCs must be deformed by shear forces before they can pass through a capillary (see Figure 1). In this case, the erythrocyte velocity in capillaries should depend mainly on the deformability of the RBCs. It has been shown in the past that rigidified RBCs struggle to pass capillaries or even plugs [50].

Loss of RBC deformability and/or increased aggregability favors the development of thrombotic processes in the microcirculation [18,52]. This has previously been described in COVID-19 patients [10,53], especially when platelets are also activated [54,55,56].

## 3. Consequences of RBC Dysfunction on the Microcirculation

**Figure 2:**
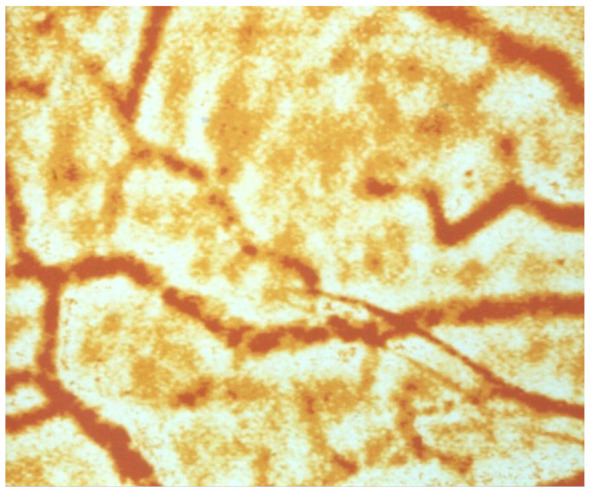
Conjunctival vessels with red blood cell aggregates that can only pass through capillaries, when the shear forces are high enough to create a single-cell flow in precapillary arterioles where the shear forces are highest [62]). magnification 1:150.

Previous studies with circulatory disorders have shown that both rigid RBCs and increased RBC aggregation are associated with slower blood flow velocities in the capillary bed [18,57,58,59]. RBCs with reduced deformability have difficulty penetrating and passing through capillaries. They usually do so in a single-file flow [60]. However, this requires that RBC aggregates—as shown in Figure 2—are disrupted by appropriate shear forces in the precapillary arterioles (where the highest shear forces in the vasculature occur) [61]. If this does not occur, such small aggregates pass very slowly through the capillary bed [62].

Recent studies have now shown that COVID-19 erythrocytes also have impaired rheological properties. Loss of deformability of the RBCs [20,21,22,46,63] and increased aggregation of RBCs have been reported [20,63]. While rigid RBCs hinder the capillary passage, increased aggregation may impede entrance into the microcirculation and by forming rouleaux or clumps may also impede flow in the postcapillary venules. In this sense, the increased aggregation may exacerbate COVID-19 microangiopathy.

Consistent with these pathological cellular alterations, which are critical for microcirculatory flow [64,65], microcirculatory disturbances have already been demonstrated. Koutsiaris showed that the RBC velocity in conjunctival microvessels of COVID-19 patients was not only significantly reduced but also had a dramatic effect on the incidence of microthrombosis despite thromboprophylaxis [11]. Abdominal microcirculatory disturbances have also been reported in severe cases of COVID-19 [10]. This is corroborated by *post mortem* studies showing the presence of microthrombi in the failing heart [66] or in the lungs [67]. Furthermore, autopsy results showed that a significant proportion of SARS-CoV-2-infected patients suffered from thromboembolic events predominantly affecting the venous system [68,69].

Increased RBC aggregation—as described, e.g., by the Connes group [20]—may also exacerbate COVID-19 microangiopathy. In this condition, a massive sludging has been described—firstly in the conjunctiva [64], and later also in human cutaneous capillaries [18]. Another study showed that large RBC aggregates—as can be found in the conjunctival vessels of diabetic patients (See Figure 2) [62]—pass very slowly through precapillary arterioles [62].

The concomitant decrease in RBC hemoglobin concentration [63] may amplify the effect of reduced oxygen carriers into the microcirculation. The oxygen content of whole blood from COVID-19 patients was significantly reduced compared to the pO2 of healthy donors, while the carbon dioxide concentration was unaffected [70].

## 4. SARSCoV-2 and Endothelial Cells

To complete the mosaic, endothelial cells play also a striking role in this scenario [71]. Endothelial cells regulate the relaxation and constriction of blood vessels, the extravasation of solutes, fluid, and hormones, as well as the blood flow [72,73]. They balance coagulation and fibrinolysis and are a major actor in the regulation of immunology, inflammation, and secondary angiogenesis. The cytokine storm increases vascular permeability leading to edema and hypoxia and promotes microvascular dysfunction [74]. The endothelial glycocalyx contributes to coagulation by releasing procoagulant factors such as the von Willebrand factor, tissue factor, thromboxane A2, and plasminogen activator inhibitor 1. In addition, anticoagulant factors such as thrombomodulin, tissue plasminogen activator (tPA), tissue factor pathway inhibitor, and prostaglandin I2 are synthesized [72]. Infection of the endothelial cells by SARSCoV-2 causes multiple phenomena that trigger a hypercoagulative state, namely, the detachment of endothelial cells, so that the sub-endothelium becomes accessible to passing platelets, contributing to thrombin generation, the release of von Willebrand factor and tissue factors, NETosis, and pyroptosis [9,75,76,77,78,79]. The former states can contribute to thromboembolic complications, whereas the latter may result in cell death [76]. Inflammation-induced endotheliitis [12,80] together with compromised blood fluidity and neutrophil extracellular trap formation have been described as being associated with microvascular thrombosis [13,80,81].

## 5. Conclusions

Alterations in the deformability and aggregability of ex vivo COVID-19 RBCs have been proven in initial studies. Such changes in erythrocytes may play a role in the impairment of capillary blood flow and the COVID-19-related microangiopathy/microthrombosis that have also already been detected *in vivo*. However, it must be emphasized that this is most likely not the only cause. Activated platelets and endothelial cells are also of considerable importance for the occlusive events in the microcirculation.

## Data Availability

No new data were created or analyzed in this study. Data sharing is not applicable to this article.
